# High-Resolution Melting Analysis for the Rapid Detection of Fluoroquinolone and Streptomycin Resistance in *Mycobacterium tuberculosis*

**DOI:** 10.1371/journal.pone.0031934

**Published:** 2012-02-21

**Authors:** Ann S. G. Lee, Danny C. T. Ong, Joshua C. L. Wong, Gilman K. H. Siu, Wing-Cheong Yam

**Affiliations:** 1 Division of Medical Sciences, National Cancer Centre, Singapore, Singapore; 2 Department of Microbiology, Yong Loo Lin School of Medicine, National University of Singapore, Singapore, Singapore; 3 Department of Microbiology, Li Ka Shing Faculty of Medicine, The University of Hong Kong, Hong Kong Special Administrative Region, China; St. Petersburg Pasteur Institute, Russian Federation

## Abstract

**Background:**

Molecular methods for the detection of drug-resistant tuberculosis are potentially more rapid than conventional culture-based drug susceptibility testing, facilitating the commencement of appropriate treatment for patients with drug resistant tuberculosis. We aimed to develop and evaluate high-resolution melting (HRM) assays for the detection of mutations within *gyrA*, *rpsL*, and *rrs*, for the determination of fluoroquinolone and streptomycin resistance in *Mycobacterium tuberculosis* (MTB).

**Methodology/Principal Findings:**

A blinded series of DNA samples extracted from a total of 92 clinical isolates of MTB were analyzed by HRM analysis, and the results were verified using DNA sequencing. The sensitivity and specificity of the HRM assays in comparison with drug susceptibility testing were 74.1% and 100.0% for the detection of fluoroquinolone resistance, and 87.5% and 100.0% for streptomycin resistance. Five isolates with low level resistance to ofloxacin had no mutations detected in *gyrA*, possibly due to the action of efflux pumps, or false negativity due to mixed infections. One fluoroquinolone-resistant isolate had a mutation in a region of *gyrA* not encompassed by our assay. Six streptomycin-resistant strains had undetectable mutations by HRM and DNA sequencing, which may be explained by the fact that not all streptomycin-resistant isolates have mutations within *rpsL* and *rrs*, and suggesting that other targets may be involved.

**Conclusion:**

The HRM assays described here are potentially useful adjunct tests for the efficient determination of fluoroquinolone and streptomycin resistance in MTB, and could facilitate the timely administration of appropriate treatment for patients infected with drug-resistant TB.

## Introduction

*Mycobacterium tuberculosis* (MTB) is the world's leading cause of mortality due to an infectious agent with global estimates of 2 billion people currently infected worldwide [1]. The WHO Report 2010 on “Global Tuberculosis Control” documents that in 2009 there were 9.4 million incident cases of tuberculosis and approximately 1.3 million deaths (http://www.who.int/tb/publications/global_report/2010/gtbr10_main.pdf). Complicating this scenario is the emergence of multidrug-resistant tuberculosis (MDR-TB), defined as resistance to two first-line antitubercular drugs, isoniazid and rifampin, causing great global concern and resulting in an increased need for the understanding of the molecular mechanisms and molecular epidemiology of drug resistance [2], [3], [4]. In 2008, there were an estimated 440,000 cases of MDR-TB, with the highest numbers of cases occurring in China, India, the Russian Federation and South Africa (http://www.who.int/tb/publications/global_report/2010/gtbr10_main.pdf).

The current standard TB treatment regimen is in two stages: two months of rifampin, isoniazid, pyrazinamide and ethambutol, followed by four months of rifampin and isoniazid. MDR-TB treatment requires use of second-line drugs such as fluoroquinolones (Moxifloxacin, gatifloxacin, levofloxacin) or injectable agents such as aminoglycosides (streptomycin, amikacin, kanamycin) and polypeptides (capreomycin) for typically two years [5], [6]. These second-line drugs are very poorly potent, highly toxic and expensive. Thus, the rapid identification of drug-resistant MTB using molecular methods could aid in more appropriate treatment given earlier, and have the potential to decrease transmission of the resistant strains. In addition, the development of a rapid, low cost and sensitive assay could potentially be used in countries with high rates of MTB and where cost effectiveness is essential.

Molecular methods for the determination of drug resistance are designed to target specific genes known to harbor mutations associated with resistance to specific anti-tuberculous drugs. Mutations associated with fluoroquinolone resistance occur in the quinolone resistance-determining regions (QRDR) of the *gyrA* and *gyrB* genes, which encode the A and B subunits of DNA gyrase, with mutations at codons 90, 91 and 94 in *gyrA* being most common [7], [8], [9], [10]. Streptomycin resistance in MTB is associated with mutations in *rpsL* and *rrs*, encoding the ribosomal protein S12 and 16S rRNA respectively [11], [12]. Within the *rpsL* gene, mutations at codons 43 and 88 have been reported in streptomycin-resistant *M. tuberculosis*, with the K43R mutation being the most common [12], [13], [14], [15]. Mutations within *rrs* have been found in the 530 loop, the 912 loop and the 1400 region, but these are less common than mutations within the *rpsL* gene [12], [13], [15].

The high-resolution melting (HRM) assay has been effectively used to detect mutations within genes associated with human diseases and in microbes, to subtype viruses, and for species differentiation [16], [17], [18], [19], [20]. It has been widely utilized for a variety of applications as it is cost-effective, rapid, sensitive, and specific [21], [22]. HRM employs saturating double-stranded DNA-binding dyes included during the PCR process and the denaturation of PCR amplicons with real-time monitoring of fluorescence, following PCR. Sequence variants are detected from differences in the melting profiles between test and reference DNA. We have previously described a HRM assay for the detection of resistance to the first-line antitubercular drugs, isoniazid and rifampicin [22]. For this current study, we aimed to develop a HRM assay to scan for mutations in *gyrA*, *rpsL* and *rrs*, for the determination of fluoroquinolone and streptomycin resistance in *Mycobacterium tuberculosis*.

## Materials and Methods

### Clinical isolates of Mycobacterium tuberculosis and drug susceptibility testing

*M. tuberculosis* clinical isolates for assay development were collected from the Central Tuberculosis laboratory, Department of Pathology, Singapore General Hospital (SGH) and were screened for drug susceptibility with the BACTEC 460 radiometric method (Becton Dickinson, Sparks, MD, USA), as described previously [13], [23], [24].

For assay validation, a blinded series of 83 DNA samples of *M. tuberculosis* were obtained from the Department of Microbiology, Li Ka Shing Faculty of Medicine, the University of Hong Kong. These isolates were tested for drug susceptibility as previously described [25], [26]. In addition, 9 blinded samples from SGH were also used for validation. Of the total of 92 blinded samples, 53 and 62 were used for validation of our fluoroquinolone and streptomycin HRM assays respectively.

### DNA extraction

DNA was extracted from the *M. tuberculosis* clinical isolates from Singapore as described previously [22], [27]. DNA from clinical isolates from Hong Kong was extracted as described [28], and were further purified using phenol-chloroform-isoamyl alcohol (25∶24∶1) (Invitrogen). DNA concentration was measured using the Nanodrop 1000 (Thermo Scientific, Waltham, MA).

### Real-time PCR and high-resolution melting analysis

Real-time PCR and high-resolution melting analysis was performed as described previously [22]. In brief, PCR was performed in 10-ul reactions containing 0.2 ng sample DNA, 0.2 ng reference DNA from *Mycobacterium tuberculosis* H37Rv, 1× PCR buffer containing 1.5 mM MgCl_2_, 200 µM dNTPs, 200 nM of each primer (Table 1), 1.5 uM Syto9 (Invitrogen), 0.5 U of HotStarTaq polymerase (Qiagen) and 4 ul of mineral oil (Sigma Aldrich) on the Rotor-Gene 6000 (Corbett Research) with the following PCR cycling parameters: 95°C for 15 minutes; 40 cycles at 95°C for 20 seconds and the appropriate annealing temperature (Table 1) for 30 seconds. The melt curve was generated by heating at increments of 0.1°C/s, using the temperature ranges shown in Table 1. The HRM curve was analyzed using the Rotor-Gene 1.7.87 software. (The Rotor-Gene is currently available from Qiagen, which has acquired Corbett Research.)

**Table 1:** Primer sequences used for fluoroquinolone and streptomycin resistance HRM detection assays.

Primer name^a^	Primer sequence	Amplicon size (bp)	Annealing temp (°C)	HRM temp range (°C)	Nucleotide positions^b^	HRM amplicon range^c^
**Fluoroquinolone**						
gyrA_F	5′-GGTGCTCTATGCAATGTTCG-3′	211	60	92 to 95	162 to 181	Codon 61 to 118
gyrA_R	5′-CGGTGGGTCATTGCCT-3′				372 to 357	
**Streptomycin**						
rpsL_F	5′-CAGCGTCGTGGTGTATGC-3′	232	60	86 to 94	85 to 102	Codon 35 to 99
rpsL_R	5′-CCTGCGTATCCAGCGAAC-3′				316 to 299	
rrs1_F	5′-ACCGGCCAACTACGTGC-3′	102	60	81 to 89	493 to 509	Nuclotide 510 to 575
rrs1_R	5′-GAACAACGCGACAAACCAC-3′				594 to 576	
rrs2_F	5′-CTAGGTGTGGGTTTCCTTCC-3′	153	60	82 to 90	817 to 836	Nucleotide 837 to 943
rrs2_R	5′-CGTTGCATCGAATTAATCCAC-3′				964 to 944	
rrs3_F	5′-TCCCGGGCCTTGTACACA-3′	62	60	80 to 87	1374 to 1391	Nucleotide 1392 to 1416
rrs3_R	5′-CCACTGGCTTCGGGTGTTA-3′				1435 to 1417	

a F: Forward, R: Reverse.

b Nucleotide position is relative to the transcriptional start site of each gene.

c Amplicon range of the HRM primers does not include the primer regions.

### DNA sequencing

Mutation screening by direct DNA sequencing was done as described previously [13], [23], [26], [29], or using the PCR primers listed in Tables 1 or 2, with sequencing performed on an Applied Biosystems 3130xl genetic analyzer.

**Table 2:** Primer sequences used to sequence *rpsL*, *rrs*, and *gyrA*.

Gene (Rv no.)	Gene ID	Primer name	Length^a^	*T_m_*	%GC^b^	Direction^c^	Sequence (5′- 3′)	Nucleotide position
***gyrA*** ** (Rv0006)**	887105	gyrA-F	20	59.3	50.0	F	GGTGCTCTATGCAATGTTCG	162 to 181
		gyrA-R	19	61.1	52.6	R	GGGATATTGGTTGCCATGC	551 to 569
***rpsL*** ** (Rv0682)**	888259	rpsL-F	20	58.6	45	F	AAAGCGCCCAAGATAGAAAG	−27 to −7
		rpsL-R	19	59.3	57.9	R	CAACTGCGATCCGTAGACC	422 to 440
***rrs*** ** (Rvnr01)**	2700429	rrs1-F	20	57.3	45	F	ATACCTTTGGCTCCCTTTTC	−17 to −36
		rrs1-R	21	59.6	57.1	R	GGAAACCCACACCTAGTACCC	811 to 831
		rrs2-F	20	59.8	55	F	GCGCAGATATCAGGAGGAAC	688 to 707
		rrs2-R	20	58.4	55	R	CGCCCACTACAGACAAGAAC	1586 to 1605

a Length, number of nucleotides.

b %GC, number of G's and C's in the primer as a percentage of the total number of nucleotides.

c F, forward; R, reverse.

### Polymorphism detection for the *gyrA* HRM assay

A natural polymorphism occurs in codon 95 (Ser-95/Thr-95) of *gyrA* [30], [31]. In order to prevent false positive mutant detection in susceptible isolates due to the presence of the *gyrA* codon 95 polymorphisms, two sets of *gyrA* HRM assays using reference DNA with either the Ser-95 or Thr-95 polymorphism were done. Genomic DNA from *Mycobacterium tuberculosis* strain H37Ra (ATCC, USA) was used as the *gyrA* Ser-95 reference DNA, and DNA from a previously sequenced fluoroquinolone-susceptible strain was used as the *gyrA* Thr-95 reference. Samples with mutations within *gyrA* will demonstrate changes in the melt curve shape for both sets of reference DNA used, but polymorphisms at codon 95 in susceptible isolates would be identified if deviations are seen in only one set.

### Sensitivity of the *gyrA* HRM assay in the detection of mixed populations

Mixed populations of different *M. tuberculosis* strains have been detected in clinical samples from pulmonary tuberculosis patients [32], [33]. To test the HRM assay for its limit in detecting mutations within mixed populations, DNA of a *gyrA* mutant strain (D94G) was titrated and mixed with DNA of a wildtype strain to artificially create samples with mixed populations. The mutant DNA was serially diluted at concentrations of 100%, 50%, 25%, 12.5% and 6.25%. Next, HRM was performed and the melting profiles were compared with a 100% wildtype reference.

### Statistical analysis

Sensitivity is defined as [Number of drug-resistant isolates with mutations]/[number of drug-resistant isolates with mutations+number of drug-resistant isolates without mutation]; and specificity as [Number of drug-susceptible isolates without mutations]/[number of drug susceptible isolates with mutations+number of drug-susceptible isolates without mutations] [22]. Calculation of the 95% confidence interval was performed using the Adjusted Wald method (http://www.measuringusability.com/wald.htm).

## Results

**Figure 1:**
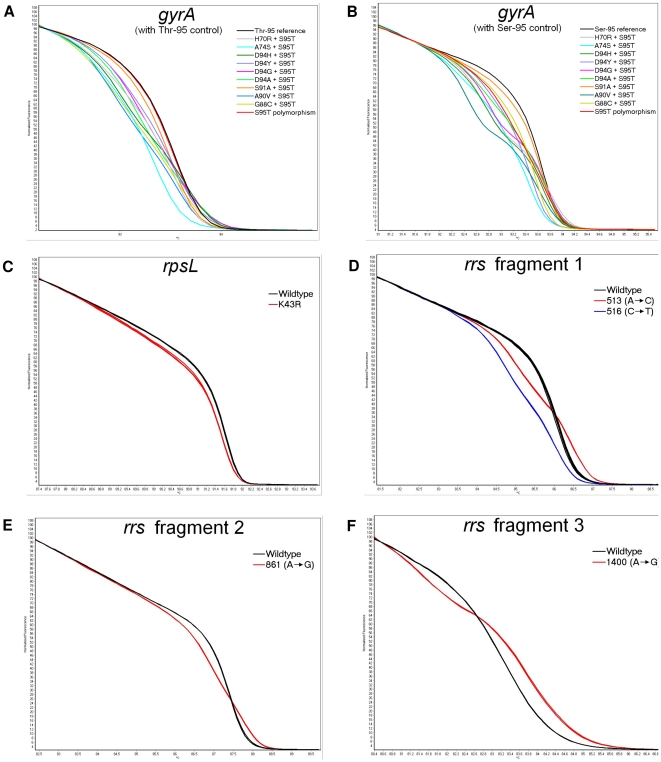
Representative high resolution melt curves of (A) *gyrA* with Thr-95 control, (B) *gyrA* with Ser-95 control, (C) *rpsL*, and (D, E, F) *rrs* fragments 1, 2, 3 respectively, demonstrating the change in melt curve shape caused by mutations. Wildtype samples are shown in black and samples with mutations are shown in color. Experiments were performed in duplicate.

Representative normalized melt curves from HRM analysis of *gyrA*, *rpsL* and *rrs* are shown in Figure 1. Samples with mutations, represented by colored lines, are easily differentiated from the susceptible (wildtype) isolates, indicated by black lines, by differences in the shape of the melt curves. Note that there is good concordance in the melting profile for the susceptible isolates, with one composite melt curve for all the susceptible isolates (including the reference DNA from MTB H37Rv), as shown by the black line.

### Determination of fluoroquinolone resistance

A blinded series of 53 MTB clinical isolates were used for the detection of fluoroquinolone resistance, by HRM analysis of the *gyrA* gene. There were phenotypic and HRM genotypic concordance for 47 of 53 isolates (Table 3). The HRM assay was successful in detecting previously reported *gyrA* mutations namely, H70R [34], A74S [26], G88C, A90V, S91A, and D94A/G/H/Y [35], [36], [37], [38], [39]. There were discrepant results for six isolates. Five isolates had no mutations detected in *gyrA* by HRM analysis and DNA sequencing, but were found to have low level resistance to ofloxacin (MICs of 4 ug/ml) [25]. One other discrepant isolate had a mutation (A126R) in *gyrA* outside the region encompassed by the HRM assays (Table 1), identified by DNA sequencing. The HRM results for mutation detection in *gyrA* were fully concordant with DNA sequencing of the region encompassed by the HRM assay.

**Table 3:** HRM results for the screening of fluoroquinolone and streptomycin resistance in blinded series of MTB samples.

Phenotype	No. of isolates	HRM and DNA sequencing results^a^	
**Fluoroquinolone**		***gyrA***	
Susceptible (28)	28	NM	
Resistant (25)	1	M [H70R (cAc→cGc)]	
	1	M [A74S (Gcc→Tcc)]	
	1	M [G88C (Ggc→Tgc)]	
	1	M [A90V (gCt→gTg)]	
	1	M [A90V (gCt→gTg)]	
	1	M [S91A (Tcg→Ccg)]	
	1	M [D94Y (Gac→Tac)]	
	1	M [D94H (Gac→Cac)]	
	2	M [D94A (gAc→gCc)]	
	9	M [D94G (gAc→gGc)]	
	1^b^	NM {A126R (GCg→AGg)}	
	5^b^	NM	
**Streptomycin**		***rpsL***	***rrs***
Susceptible (14)	14	NM	NM
Resistant (48)	28	M [K43R (aAg→aGg)]	NM
	1	M [K43R (aAg→aGg)]	M [nt.861 (A→G)]
	1	M [K43R (aAg→aGg)]	M [nt.1400 (A→G)]
	1	M [K43R (aAg→aGg)]	M [nt.513 (A→C)]
	1	M [K43R (aAg→aGg)]	M [nt.516 (C→T)]
	5	N/A	M [nt.513 (A→C)]
	5	N/A	M [nt.516 (C→T)]
	1^b^	NM {K121K (aaA→aaG)}	NM
	1^b^	NM {K121K (aaA→aaG)}	{nt.15 (T→C)}
	4^b^	NM	NM

a HRM results are represented as “M” for mutant and “NM” for non-mutant. Mutations detected by DNA sequencing are represented in square brackets [ ], or by curly brackets {} if the mutations are in regions not covered by the HRM assay.

b Isolates with discrepant HRM results as compared to their respective drug resistant phenotypes.

To specifically identify a polymorphism at codon 95 of *gyrA*, two separate HRM experiments with either Ser-95 reference DNA or Thr-95 reference DNA were performed. Figure 1A, using the Thr-95 reference DNA showed no change in melting curve profile for a non-mutant fluoroquinolone susceptible isolate with the S95T polymorphism, however, the same isolate showed a deviation in melt curve shape when Ser-95 reference DNA was used (Figure 1B), thus clearly identifying this isolate as not being mutated. In contrast, mutant samples had deviations in their melt curve shapes for both sets of experiments (Figure 1A and 1B).

### Determination of streptomycin resistance

HRM analysis of *rpsL* and *rrs* for the detection of streptomycin resistance was performed on a blinded series of 62 *M. tuberculosis* clinical isolates (Figure 1C–1F; Table 3). Phenotypic and HRM genotypic determination of streptomycin susceptibility was concordant for 56 of 62 isolates (Table 3). Six streptomycin-resistant isolates did not have any detectable mutations using HRM analysis of *rpsL* and *rrs*. Importantly, DNA sequencing of the regions encompassed by the HRM assay showed concordance with the HRM assay results for these isolates.

To determine if mutations not within the regions encompassed by the HRM assay may be present in the two isolates with discrepant results, we sequenced *rpsL* and *rrs* in entirety. Results from the sequencing showed a single *rpsL* mutation at position 363 (A→G) which encodes for K121K, in both samples. There was a single alteration (T→C) at position 15 in *rrs* in one sample, whereas the other sample had no mutations in *rrs*.

To determine if the *rpsL* K121K and the T→C alteration at position 15 of *rrs* are present in other streptomycin-susceptible isolates, 8 isolates were sequenced. All 8 streptomycin-susceptible isolates had the *rpsL* K121K alteration but not the T→C alteration at position 15 of *rrs*.

One isolate that was streptomycin-susceptible by phenotype, had initially been found to be mutation-positive by HRM analysis, but repeat testing on a re-extracted DNA sample showed no mutations by HRM analysis. The initial discrepant result between drug susceptibility and HRM testing may have been due to contamination of the culture with a mutant strain, as subsequent re-culture and re-extraction of that sample revealed no mutations.

### Limit of detection of mutants in mixed populations

**Figure 2:**
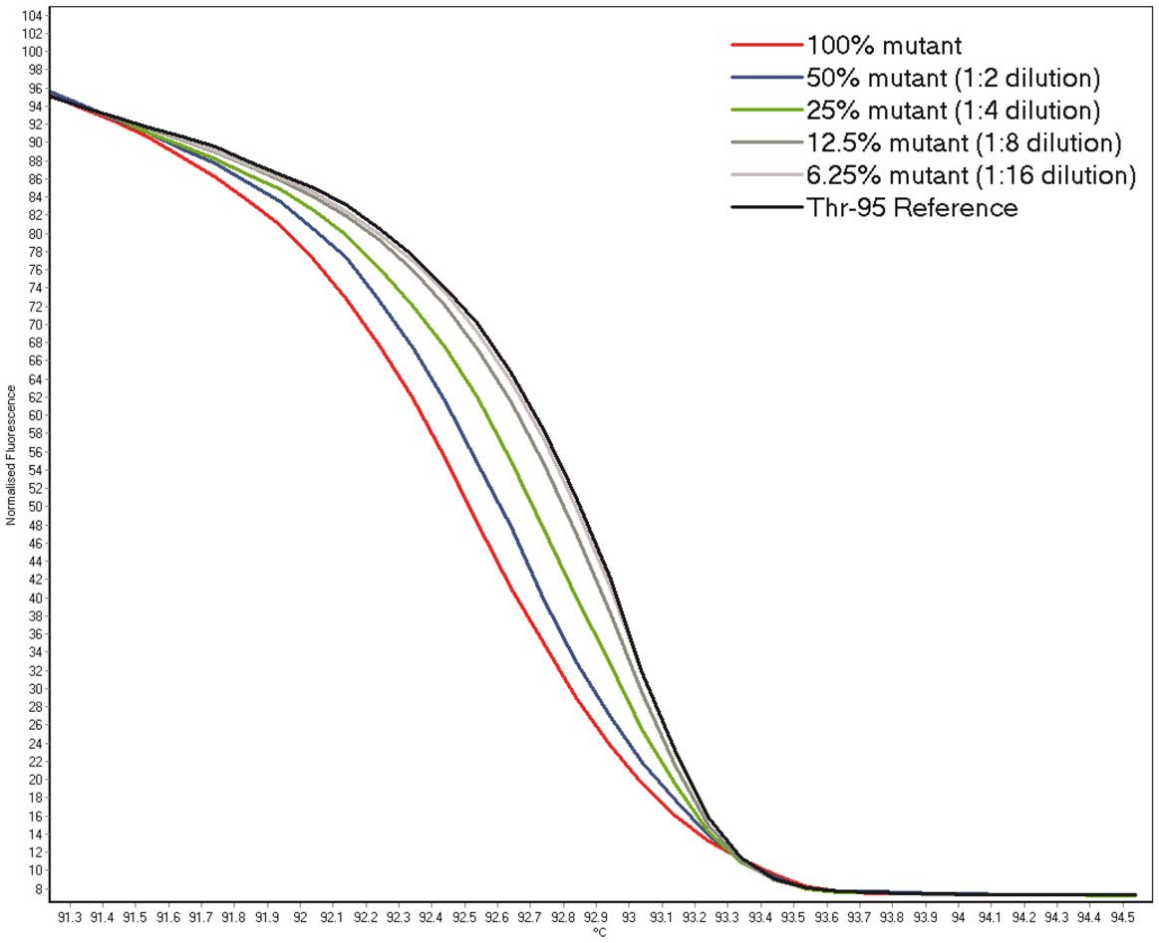
High resolution melt curves of a mutant DNA sample (*gyrA* D94G) serially diluted at concentrations of 100%, 50%, 25%, 12.5%, and 6.25%, and mixed with wildtype DNA. Wildtype samples are shown in black and samples with mutations are shown in color. Changes in melt curve shape demonstrating the presence of mutations were observed in samples with 100%, 50% and 25% mutant DNA. Experiments were performed in duplicate.

Titration of *gyrA* mutant (D94G) and wildtype DNA samples, followed by HRM showed that mutations in samples are detectable at a threshold limit of 25% (Figure 2).

### Sensitivity and specificity

The sensitivity of the HRM assays for the detection of fluoroquinolone and streptomycin resistance were 74.1% and 87.5% respectively, and the specificity values were 100.0% and 100.0% respectively (Table 4). The HRM assay was directly compared to the conventional phenotypic assay to calculate the sensitivity and specificity.

**Table 4:** Sensitivity and specificity of the drug resistance detection HRM assays.

Drug susceptibility	No. of isolates		Sensitivity^a^ (95% CI^b^)	Specificity^c^ (95% CI^b^)
	Mutation positive by HRM	Mutation negative by HRM		
**Fluoroquinolone (n = 53)**			19/25, 74.1%	28/28, 100.0%
Resistance	19	6	(56.3—88.8)	(89.5—100.0)
Susceptible	0	28		
**Streptomycin (n = 62)**			42/48, 87.5%	14/14, 100.0%
Resistance	42	6	(74.9—94.5)	(80.9—100.0)
Susceptible	0	14		

a [Number of drug-resistant isolates with mutations]/[number of drug-resistant isolates with mutations+number of drug-resistant isolates without mutation].

b Statistical calculations were performed with the free software available from http://www.measuringusability.com/wald.htm using the Adjusted Wald method.

c [Number of drug-susceptible isolates without mutations]/[number of drug-susceptible isolates with mutations+number of drug-susceptible isolates without mutation].

## Discussion

Drug resistance in tuberculosis is currently determined using culture-based methods such as the agar proportion method, or liquid media methods like the BACTEC MGIT 960 which are more rapid but which still require at least one week for the determination of drug susceptibility [40], [41]. In order to expedite the determination of fluoroquinolone and streptomycin resistance in MTB, we have developed HRM assays for the detection of mutations in *gyrA*, *rpsL* and *rrs*, and have assessed the performance of these assays using blinded series of DNA samples from drug-resistant and –susceptible MTB clinical isolates.

There was a 100% concordance between the HRM assays and DNA sequencing for all genes analyzed in this study. These HRM assays are thus accurate, in addition to being easy to implement, rapid and cost-effective [22]. The low cost of this assay, estimated at US$0.30 per HRM reaction [22] would be an advantage in countries where cost-effectiveness is important and where MTB infection is prevalent, such as in India and China.

However, the disadvantage of using the HRM assay is that multiple amplicons may have to be designed, especially for larger genes like *rrs*, since differences in melt curve shape are more easily discriminated with smaller amplicons. In patients infected with mixed bacterial populations [32], [33], our *gyrA* assay has a limit of detection of mutants of 25%, which may result in false negative results for some patients. For genes such as *gyrA*, which has a natural polymorphism within the region analyzed for HRM, two sets of experiments need to be done, using reference DNA for both alleles of the polymorphism. As mentioned in our previous publication [26], the S95T polymorphism exists in all Beijing strains, and hence in geographical regions where Beijing strains are predominant, ofloxacin-susceptible clinical strains with the S95T polymorphism should be selected as the reference for the first HRM assay. Only samples shown to have deviations in melt curve shape in this first set of experiments need to be run in the second set of experiments using Ser-95 (H37Ra) as reference.

Our HRM assay detected mutations within the *gyrA* gene in 19 of 25 (74.1%) fluoroquinolone-resistant isolates. Of the six resistant isolates with no detectable mutations by HRM analysis, two isolates had mutations in regions not encompassed by our assay, while four isolates with low level resistance had no detectable mutations within *gyrA*. It has been suggested that the mechanism for resistance in such isolates may be mediated by active efflux pumps, as *in vitro* studies have shown that the use of efflux pump inhibitors resulted in the reduction of MIC levels [42], [43], [44].

The HRM assay for the detection of streptomycin-resistance had a sensitivity and specificity of 87.5% and 100.0% respectively. However, discrepant results between phenotyping and molecular assays were obtained for two isolates which were streptomycin-resistant by phenotype, but with no alterations detected by HRM. DNA sequencing of the entire *rpsL* and *rrs* for these isolates revealed alterations not within the region encompassed by the HRM assay. The K121K alteration (AAA→AAG) in *rpsL*, which has previously been reported, was detected in both isolates [29], [45], [46]. Niemann *et al.* (2009) has suggested the possibility of a sequencing error in the original H37Rv sequence obtained from PubMed (NCBI) resulting in this K121K “alteration” [47]. Sequencing of an additional eight streptomycin-susceptible isolates revealed the K121K “alteration” in all samples, supporting Niemann's observation, and suggesting that it is a polymorphism. In addition, DNA sequencing detected a T-to-C alteration at position 15 in *rrs* in one sample that has not been previously reported in streptomycin-resistant isolates, and which could possibly be a novel mutation associated with resistance. This finding warrants further investigation on additional streptomycin resistant and susceptible isolates from other geographical locales.

Although mutations in *rpsL* and *rrs* are known to be associated with streptomycin resistance, not all resistant isolates have mutations in these genes. A higher frequency of mutations in *rpsL* and *rrs* has been observed in geographical areas with a high prevalence of Beijing strains, for example in Singapore, Latvia and Japan [13], [14], [15]. However, in India, Rwanda and Yemen, none to 20% of streptomycin resistant isolates had mutations in *rpsL* or *rrs* [12], [29]. This thus suggests that other additional genes may be involved in streptomycin resistance, for instance the *gidB* gene that encodes a 7-methylguanosine methytransferase specific for 16S rRNA [48]. Although mutations in the *gidB* gene have been shown to confer low-level streptomycin resistance, mutations in streptomycin-susceptible clinical isolates have also been detected, and thus further investigations are warranted to confirm the association of *gidB* with streptomycin resistance [49], [50].

The sensitivity of a molecular assay depends on the frequency of detectable mutations within genes associated with resistance, in resistant isolates. For example, mutations within the rifampicin resistance-determining region (RRDR) of the *rpoB* gene, occur at frequencies of >95% in rifampicin-resistant MTB isolates [51], [52] and thus molecular assays targeting *rpoB* would have a similarly high sensitivity. In contrast, resistance associated mutations occur at frequencies of 54.8 to 90% at the QRDR of *gyrA* in fluoroquinolone-resistant isolates [53], [54], and 0 to 95% in the *rpsL* and *rrs* genes in streptomycin-resistant isolates [12], [13], [14], [15], [29]. Not all resistant isolates have detectable mutations in genes currently known to be associated with specific drug resistance and therefore, in order to improve the sensitivity of molecular tests, it will be necessary to discover additional novel genes associated with resistance.

The HRM assays described here are potentially useful adjunct tests for the rapid detection of fluoroquinolone and streptomycin resistance in MTB, and could facilitate the timely administration of appropriate treatment for patients with drug-resistant TB.
